# Stability of end-of-life preferences in relation to health status and life-events: A cohort study with a 6-year follow-up among holders of an advance directive

**DOI:** 10.1371/journal.pone.0209315

**Published:** 2018-12-18

**Authors:** Matthijs P. S. van Wijmen, H. Roeline W. Pasman, Jos W. R. Twisk, Guy A. M. Widdershoven, Bregje D. Onwuteaka-Philipsen

**Affiliations:** 1 Department of Public and Occupational Health, Amsterdam Public Health research institute, VU University Medical Center, Amsterdam, The Netherlands; 2 Department of Epidemiology and Biostatistics, VU University Medical Center, Amsterdam, The Netherlands; 3 Department of Medical Humanities, Amsterdam Public Health research institute, VU University Medical Center, Amsterdam, The Netherlands; Boston University School of Medicine, UNITED STATES

## Abstract

**Background:**

Stating preferences about care beforehand using advance care planning and advance directives has become increasingly common in current medicine. There is still lack of clarity what happens over the course of time in relation to these preferences. We wanted to determine whether the preferences about end-of-life care of a person owning an advance directive stay stable after the experience of a life-event; how often advance directives are altered and discussed with family members and physicians over time.

**Design:**

A longitudinal cohort study with a population consisting of people owning the most common advance directives in the Netherlands, with a follow-up of 6-years from 2005 until 2011. Respondents were recruited using two associations that provided the advance directives, Right to Die-NL (n = 4463) and the Dutch Patient Organisation (n = 1263). Each 1.5 year a questionnaire was sent. We analyzed the relationship between variables using generalized estimated equations.

**Results:**

96.9–98.1% of the respondents who had experienced a life-event had stable preferences. 89.9–93.7% of Right-to-Die-NL-members who had experienced a life-event didn’t make any alterations in their advance directives. During the 6-year course of our study, a minority of both groups didn’t discuss their advance directive with anyone (8.7–16.4%), while a majority didn’t discuss it with physicians (ranging 58.1–95.1%). Factors related to health, such as deterioration in experienced health, increased the odds to discuss advance directives.

**Conclusion:**

Our results largely dispute criticism concerning usability of advance directives due to lack of stability of preferences. Whereas a change in health status and the experience of other life-events were not related to instability in preferences, they did increase the odds of communication about advance directives.

Because our results show that the possession of an advance directive does not necessarily result in frequent discussions between patients and caregivers, a more structured approach like advance care planning might be a solution.

## Introduction

Stating preferences about care beforehand using advance care planning and advance directives has become increasingly common in current medicine. Advance directives (ADs) are instruments aimed at ensuring medical care at the end of life according to someone’s wishes in case of incompetence. Yet, it is unclear what happens over the course of time in relation to these documents. Are the preferences they are supposed to represent stable, also after changes in health status of their owners or the experience of other life-events? Do their owners make alterations in their ADs? How often are these documents discussed, and with whom?

Auriemma et al. did a systemic review on stability of preferences of mainly patients and older adults [1]. They found that preferences overall remain stable, with higher stability among patients suffering from a serious illness [1,2] and people who had completed an advance directive [3–7]. However, they also stress the need for further research on this subject, especially on the relation between stability of preferences and a change in health status, because findings until now on this subject are inconclusive. Besides a change in a person’s health status, other type of life-events could also be relevant when it comes to stability of preferences. An indication for this could be our finding in a previous study that the experience of someone suffering a serious illness or a death in a person’s close surroundings was linked to the motivation to draw up an AD [8]. While there is data on stability of preferences, it is not known whether owners of ADs make alterations in the actual documents over the course of time.

Next to stability of preferences, communication about ADs is a relevant issue. In recent years it has become increasingly clear that an AD in itself is not enough, and that it is crucial to talk about preferences. This resulted in a focus on advance care planning (ACP), a process in which a person’s preferences are discussed with a caregiver and recorded. Research showed that ACP can improve care at the end of life [9]. Because communication seems to be pivotal for the success of ADs, communication surrounding ADs over time is an important issue for research.

With the study presented in this paper we aimed to fill in some of the blanks on the subjects of stability of preferences and communication by following a cohort of people who possess the most common ADs in the Netherlands for six years. We identified people who experienced a change of health, but also other life-events, and observed if this affected their preferences about the end of life. Besides stability of preferences, we also investigated whether they actually made alterations in their ADs. Finally we looked how often ADs were discussed, with whom and what were factors associated with communication.

## Methods

This paper draws on a cohort study on people owning an AD, which took place in the Netherlands [10]. Only 7% of the Dutch population owns an AD [11,12]. Their use is not promoted by any policy. Dutch physicians are obligated by law to adhere to the wishes put down in an AD, but at the same time they can determine if a treatment at the end of life is medically futile. In practice most decisions about continuing or forgoing care at the end of life are taken with physicians, patients and their family involved [13].

In order to include people with an AD in the cohort, we used two associations who publish the most common standard formats for ADs in the Netherlands. Right to Die-NL (Nederlandse Vereniging voor een Vrijwillig Levenseinde (NVVE) in Dutch), an association that makes an effort for patient autonomy and a self-chosen end of life, provides the most common types of ADs in the Netherlands. Among them a refusal of treatment document (ROTD), a do not resuscitate order (DNR), the appointment of a healthcare proxy and an advance euthanasia directive (AED).

The second association, the Dutch Patient Association (translated from Nederlandse Patiënten Vereniging (NPV) in Dutch), is Christian orientated and provides one type of AD: the ‘wish-to-live statement’. The content of this AD states that its owner wants to receive proper care, meaning no excessive, medical futile treatments at the end of life, but also no actions with the purpose to actively end life.

The membership files of these two associations were used to recruit a random sample of respondents for the cohort. They were given extensive information about the study before they agreed to participate. The return of a completed questionnaire was taken as consent to participate. Individual participants were followed over time by repeatedly sending them questionnaires and identifying them with the use of pseudonymous identification numbers. The researchers only received anonymized data, leaving names and addresses under sole control of the associations. The Medical Ethics Review Committee of the VU University Medical Center approved this study.

### Population and questionnaires

The respondents of the cohort answered questionnaires in Dutch every 1,5 year, the first sent in 2005. These questionnaires contained questions about background characteristics as well as about more specific topics surrounding end of life care and ADs. For this paper respondents were included who indicated they possessed a completed AD at the start of the cohort in 2005 (4463 NVVE-members and 1263 NPV-members). During the progress of the study respondents dropped out of the cohort, because they deceased, moved, ended their membership with the association, or choose no longer to participate. The response rates per wave of questionnaires ranged from 82–87% for NVVE-members and 84–89% for NPV-members. Due to financial en practical reasons it was not possible for us to extend the cohort after 2011.

For previous studies we compared the members of the cohort with the Dutch general public [10,14]. We found that NVVE-respondents were more often single, higher educated and non-religious, while amongst NPV-respondents there were a lot more Protestant-Christians compared to the Dutch public. When it came to their views on the end of life, NVVE-members were similar to the general public, but more outspoken. NPV-members differed from the general public because they more often preferred to continue medical treatment at the end of life. Because of the differences between the two groups in terms of religiosity and views on the end of life, we choose to perform analyses (and present the results) for both groups separately or correct for membership of association (NVVE or NPV) when performing analyses on the population as a whole.

The questions we focused on for this paper, about preferences, the experience of life-events, adjustments in ADs and communication, were included four times in the questionnaires from the second questionnaire in 2007 till the last in 2011.

The question about stability of preferences we used for this paper was only answered in connection to the questions about the experience of life-events. We asked if the respondents experienced a life-event in the 1,5 years between this questionnaire and the previous one. The question went as follows: ‘Could you indicate if you experienced one of the following events the past one and a half year?’ Followed by captions like ‘Health (both mental or physical, for instance the diagnosis, treatment or recovery of a disease)’ and then options like ‘A change in my own health’. Respondents could choose from the following options: a change in their own health or the health of a loved-one, the death of a loved-one, the birth of (grand)child or having a new partner. The last two were combined in the experience of a positive life-event for the analyses. Respondents also had the opportunity to describe a life-event that was not amongst the given options. If respondents indicated they had experienced a life-event the pas 1,5 years, the follow-up question was if this had led to changes in their preferences.

To measure experienced quality of life we used the Eq5d, a set of questions mainly focused on health status, used to calculate a utility score. [15,16]

The subject of adjustments in ADs or the completion of new ADs during the course of the study could only be investigated with NVVE-members, while the fixed nature of the AD of the NPV did not allow alterations.

A translated version of the questionnaire used is added as a supplementary file.

### Statistical analyses

We performed descriptive statistics for the main outcome variables used for this paper, which were stability of preferences about the end of life, alterations made in ADs and how often respondents discussed their AD. We then analyzed which factors were statistically associated with making alterations in ADs and communication about them. We looked at background characteristics (gender, age, marital status, the presence of offspring, place of residence, education and religion) several variables representing (a change in) health status, and the experience of life-events.

To analyze above mentioned relationships, we used generalized estimated equations (GEE), which is suited for longitudinal analyses, because it takes into account the fact that multiple observations of one subject are used over time and corrects for within-subject correlation. To analyze the relationship between adjustments in ADs as dependent variable and background characteristics, (a change in) health status and the experience of life-events as independent variables, first univariable analyses were performed. We then put all independent variables that were significantly associated in a multivariable model. We removed variables with the highest P-values until only variables with a P-value below 0.05 remained in the model. We did the same for communication about ADs as dependent variable. Regarding instability of preferences we only performed descriptive analysis, because the amount of subjects with instable preferences was too small to perform these analyses well.

## Results

### Respondent characteristics

Table 1 shows the characteristics of the respondents of the cohort. In 2007, 63% of the NVVE-members experienced a life-event, of whom 43% experienced a change in own health, 34% a change in the health of a loved-one and 27% the death of a loved-one. Of the 66% of NPV-members who experienced a life-event, 41% experienced a change in own health, 42% a change in health of a loved-one and 41% a death of a loved-one. The answers to the questionnaires from other years showed similar results.

**Table 1:** Respondent characteristics.

Characteristic (measured in 2005, unless reported differently)	NVVE-members (n = 4463)	NPV-members (n = 1263)
**Age**, mean (SD), in years	68.3 (12.4)	59.5 (17.7)
**Sex**, No. (rounded %)		
- Male	1577 (36%)	493 (40%)
- Female	2832 (64%)	745 (60%)
**Marital status**		
- Married	2171 (50%)	844 (68%)
- Living together	247 (6%)	8 (1%)
- Partner otherwise	195 (4%)	18 (2%)
- Divorced	341 (8%)	35 (3%)
- Widowed	1057 (24%)	159 (13%)
- Single otherwise	377 (9%)	173 (14%)
**Children**		
- Children, good relation	2940 (67%)	838 (68%)
- Children, bad relation with some or all	393 (9%)	102 (8%)
- No children	993 (23%)	269 (22%)
- Otherwise	54 (1%)	20 (2%)
**Residing in (2007)**		
- Own home	3494 (96%)	1038 (96%)
- Otherwise (e.g. Nursing-home, sheltered living)	158 (4%)	45 (4%)
**Education**		
- Elementary or basic vocational	712 (16%)	436 (35%)
- Secondary	1373 (31%)	422 (34%)
- Higher	2290 (52%)	375 (30%)
**Belief and its importance in someone’s life**		
- Important belief	861 (20%)	1212 (97%)
- Not important belief or no belief	3464 (80%)	32 (3%)
**Suffering from a disease**		
- No	1473 (34%)	567 (46%)
- Yes	2916 (66%)	659 (54%)
Change in experienced health*(2007)		
- No Change	2245 (61%)	722 (66%)
- Better	186 (5%)	68 (6%)
- Much better	154 (4%)	37 (3%)
- Worse	952 (26%)	232 (21%)
- Much worse	151 (4%)	33 (3%)
**Life-events (2007)**		
Any life-event	2221 (63%)	725 (66%)
Of which**:		
- Change in own health	945 (43%)	294 (41%)
- Change in health of a loved-one	753 (34%)	304 (42%)
- Death of a loved-one	596 (27%)	295 (41%)
- Postive life-event: birth of a (grand)child or a new partner	441 (20%)	303 (42%)

* Respondents were asked how their health status was as compared to 1,5 years before.

** Percentages presented here represent the proportion of specific life-events from the total of life-events experienced.

### The experience of life-events, end-of-life preferences and adjustments in ADs

After experiencing a life-event, the preferences about the end of life of a vast majority of respondents remained unchanged or became stronger in both groups, ranging from 96.8% to 97.5% for the NVVE and from 97.4 to 98.1% for the NPV for the different intervals between questionnaires (Table 2). Over the four consecutive waves of questionnaires constantly small percentages indicated they were in doubt (ranging 1.4–1.9% for the NVVE and 1.1–1.6% for the NPV) and an even smaller percentages reported that their preferences had changed (ranging 1.2–1.6% for the NVVE and 0.4–1.1% for the NPV). Using GEE we tested if there were significant differences between the different waves when it came to instability of preferences, but there weren’t any (p = 0.514 for the NVVE, p = 0.932 for the NPV and p = 0.608 for both groups together). Because of the small percentages of instable preferences, it was not possible to analyze in what way different factors were associated with instability of preferences.

**Table 2:** Changes in preferences and ADs of people who experienced a life-event in the previous 1,5 year.

	Spring 2007	Autumn 2008	Spring 2010	Autumn 2011
**NVVE**	n = 2221	n = 2063	n = 1885	n = 1645
*Did your views or preferences about the end of life change*?				
No change or they became stronger	1815 (97.2%)	1711 (96.8%)	1797 (97.5%)	1561 (96.9%)
I’m in doubt	30 (1.6%)	34 (1.9%)	25 (1.4%)	25 (1.6%)
They changed	22 (1.2%)	23 (1.3%)	22 (1.2%)	25 (1.6%)
*Adjustments in existing AD or new AD*?*				
	198 (9.2%)	124 (6.3%)	138 (7.5%)	156 (10.1%)
**NPV**	n = 725	n = 597	n = 554	n = 482
*Did your views or preferences about the end of life change*?				
No change or they became stronger	550 (97.7%)	495 (97.4%)	527 (97.8%)	462 (98.1%)
I’m in doubt	9 (1.6%)	8 (1.6%)	6 (1.1%)	7 (1.5%)
They changed	4 (0.7%)	5 (1.0%)	6 (1.1%)	2 (0.4%)

* The subject of adjustments in ADs or the completion of new ADs during the course of the study could only be investigated with NVVE-members, while the fixed nature of the AD of the NPV didn’t allow alterations in it or the addition of new ADs besides it.

When asked if they made adjustments to their existing ADs or if they drafted a new AD during the one and a half year previous to the questionnaire, a part of NVVE-members answered they did, ranging 6.3–10.1% for the four waves of questionnaires. These differences in percentages of respondents who made adjustments that were found between the four waves of questionnaires were statistically significant when analyzed with GEE (p<0.001).

We found that only the experience of the death of a loved one remained significant in a multivariable model (OR = 1.3; 95%-CI 1.1–1.6), when we analyzed whether the experience of a life-event was statistically significantly associated with making adjustments in an AD or a new AD over time. Other variables that remained significant were higher age, having a bad relationship with some or all of your children, a higher education and an experienced health that was less than good (Table 3).

**Table 3:** Factors associated with adjustments in ADs or a new AD (only NVVE). (n = 3980, OR’s, 95%-CI’s).

Variable	univariable	multivariable
**Gender**		
- Male	1.0	
- Female	1.0 (0.9–1.2)	
**Age** (per year)	1.02 (1.01–1.02)	1.01 (1.01–1.02)
**Marital status**		
- Married	1.0	
- Living together	1.1 (0.8–1.6)	
- Partner otherwise	1.0 (0.7–1.5)	
- Divorced	1.3 (1.0–1.7)	
- Widowed	1.3 (1.1–1.6)	
- Single otherwise	1.1 (0.8–1.6)	
**Children**		
- Children, good relation	1.0	1.0
- Children, bad relation with some or all	1.5 (1.2–1.8)	1.4 (1.1–1.7)
- No children	1.1 (0.9–1.3)	1.2 (1.0–1.4)
- Otherwise	1.2 (0.7–2.0)	1.0 (0.6–1.9)
**Residing in**		
- Own home	1.0	
- Otherwise (e.g. Nursing-home, sheltered living)	1.4 (1.0–1.8)	
**Education**		
- Higher	1.0	1.0
- Secondary	0.9 (0.8–1.1)	0.8 (0.7–1.0)
- Elementary or basic vocational	0.8 (0.6–1.0)	0.7 (0.5–0.9)
**Belief and its importance in someone’s life**		
- Important belief	1.0	
- Not important belief or no belief	0.9 (0.7–1.1)	
**Experienced health**		
- Very good	1.0	1.0
- Good	1.1 (0.9–1.3)	1.0 (0.8–1.3)
- Less than good	1.8 (1.4–2.2)	1.6 (1.3–2.1)
**Suffering from a disease**		
- No	1.0	
- Yes	1.4 (1.2–1.6)	
Experienced quality of life (EQ5D tariff)*		
Per 10% decrease in score	1.10 (1.06–1.13)	
**Change in experienced health**		
- No Change	1.0	
- Better	0.9 (0.6–1.2)	
- Much better	1.3 (0.9–1.9)	
- Worse	1.5 (1.2–1.7)	
- Much worse	2.1 (1.6–2.8)	
**Life-events**		
Did not experience life-event	1.0	
Change in own health	1.3 (1.1–1.5)	
Change in health of a loved-one	1.2 (1.1–1.5)	
Death of a loved-one	1.4 (1.1–1.5)	1.3 (1.1–1.6)
Positive life-event	0.8 (0.7–1.1)	
Any life-event	1.4 (1.2–1.6)	

*The EQ5D is a validated set of questions on five domains (mobility, self-care, usual activities, pain and discomfort, anxiety and depression), which can be used to calculate a single index value representing someone’s perceived quality of life. A score of 100% represents full health and a score of 0 represents death.

### Communication about ADs

When asked with whom they had spoken about their AD the previous one and a half year, NVVE-members most often indicated their partner (ranging from 49.8% to 58.0% for the four intervals between the questionnaires), followed by their children (ranging from 33.3% to 42.4%), and general practitioner (GP, ranging from 13.7% to 22.2%; Table 4). The answers from the NPV-members showed similar results, with lower percentages: talking with their partner ranged from 30.9% to 44.8%; with their children from 17.2% to 27.3%; with their GP from 4.4% to 15.5%.

**Table 4:** Talked about AD?

	Autumn 2005- spring 2007		Spring 2007- autumn 2008		Autumn 2008- spring 2010		Spring 2010- autumn 2011		Autumn 2005- spring 2011**	
	NVVE (n = 3713)	NPV (n = 1105)	NVVE (n = 3510)	NPV (n = 1027)	NVVE (n = 3016)	NPV (n = 915)	NVVE (n = 2843)	NPV (n = 825)	NVVE (n = 2363)	NPV (n = 748)
With partner*	1150 (53.6%)	326 (42%)	987 (49.8%)	324 (44.8%)	973 (58.0%)	265 (40.8%	885 (56.1%)	179 (30.9%)	1155 (81.6%)	389 (71.9%)
With Children*	1043 (37.4%)	220 (25.9%)	876 (33.3%)	217 (27.3%)	966 (42.4%)	166 (23.3%)	862 (40.6%)	113 (17.2%)	1196 (67.5%)	259 (44.4%)
With other family members	262 (7.1%)	73 (6.6%)	226 (6.4%)	68 (6.6%)	244 (8.1%)	50 (5.5%)	203 (7.1%)	34 (4.1%)	429 (18.2%)	116 (15.5%)
With GP	609 (16.4%)	107 (9.7%)	480 (13.7%)	159 (15.5%)	619 (20.5%)	101 (11.1%)	632 (22.2%)	36 (4.4%)	991 (41.9%)	209 (27.9%)
With medical specialist	92 (2.5%)	18 (1.6%)	85 (2.4%)	17 (1.7%)	101 (3.3%)	17 (1.9%)	95 (3.3)	13 (1.6%)	160 (6.8%)	37 (4.9%)
With Others	601 (16.2%)	91 (8.2%)	547 (15.6%)	102 (9.9%)	609 (20.2%)	97 (10.6%)	544 (19.1)	74 (9.0%)	917 (38.8%)	170 (22.7%)
With no one	1334 (35.9%)	504 (45.6%)	1191 (33.9%)	417 (40.6%)	972 (32.2%)	413 (45.1%)	817 (28.7%)	474 (57.5%)	205 (8.7%)	123 (16.4%)

*Respondents who reported to have a partner or children at the time they answered the questionnaire that year.

** In order to prevent a distorted image of the data, we only could include the figures of the respondents who had answered this question in all 4 waves of the study.

A substantial part of the respondents did not speak with anyone about their AD in the preceding one and a half year period (percentages ranging 28.7–35.9% for the NVVE and 40.6–57.5% for the NPV).

When analyzing the period as a whole, from 2005 till 2011, a majority talked to somebody about their AD as least once (91.3% of the NVVE-members and 83.6% of the NPV-members). For the whole period, the results show the same order as for the four separate one-and-a-half year periods when it comes to with whom respondents talked most frequently: 81.6% of NVVE-members and 71.9% of NPV-members talked at least once with their partner during the 6 year period, 67.5% (NVVE) and 44.4% (NPV) with their children, 41.9% (NVVE) and 27.9% (NPV) with their GP (Table 4).

Almost all factors investigated were significantly associated with communication about ADs univariably, but a smaller number remained so when put in a multivariable model (Table 5). Suffering from a disease as well as a deterioration in experienced health both increased the odds of talking about ADs with family or a physician. The same was found for residing in a care-facility and experiencing the life-events of a change in own health and change in health or death of a loved-one.

**Table 5:** Factors associated with communicating about ADs. (n = 5111, OR’s, 95%-CI’s).

	Talked with family*		Talked with physician*	
**Variable**	univariable	multivariable	univariable	multivariable
**Gender**				
- Male	1.0	1.0	1.0	
- Female	1.0 (0.9–1.1)	1.2 (1.1–1.3)	1.1 (1.0–1.2)	
**Age** (per year)	1.00 (1.00–1.01)		1.03 (1.03–1.04)	1.02 (1.02–1.03)
**Marital status**				
- Married	1.0	1.0	1.0	
- Living together	1.4 (1.2–1.8)	1.5 (1.2–1.9)	1.0 (0.8–1.3)	
- Partner otherwise	1.1 (0.9–1.4)	1.2 (0.9–1.4)	1.0 (0.7–1.3)	
- Divorced	0.6 (0.5–0.7)	0.5 (0.4–0.6)	1.5 (1.2–1.8)	
- Widowed	0.8 (0.7–0.8)	0.6 (0.5–0.6)	1.7 (1.5–2.0)	
- Single otherwise	0.3 (0.3–0.3)	0.4 (0.3–0.5)	1.2 (1.0–1.5)	
**Children**				
- Children, good relation	1.0	1.0	1.0	
- Children, bad relation with some or all	0.9 (0.8–1.1)	0.9 (0.8–1.0)	1.4 (1.2–1.6)	
- No children	0.5 (0.4–0.5)	0.5 (0.5–0.6)	0.9 (0.8–1.0)	
- Otherwise	0.7 (0.5–1.0)	0.7 (0.5–0.9)	1.0 (0.7–1.5)	
**Residing in**				
- Own home	1.0	1.0	1.0	1.0
- Otherwise (e.g. Nursing-home, sheltered living)	1.2 (1.0–1.4)	1.3 (1.1–1.6)	2.0 (1.7–2.4)	1.3 (1.0–1.5)
**Education**				
- Higher	1.0		1.0	1.0
- Secondary	1.0 (0.9–1.1)		0.8 (0.7–1.0)	0.8 (0.7–0.9)
- Elementary or basic vocational	0.9 (0.8–1.0)		1.0 (0.8–1.1)	0.9 (0.8–1.1)
**Belief and its importance in someone’s life**				
- Important belief	1.0	1.0	1.0	
- Not important belief or no belief	1.4 (1.3–1.5)	0.9 (0.8–1.0)	1.3 (1.2–1.5)	
**Memberschip of association**				
- Member of NVVE	1.0	1.0	1.0	1.0
- Member of NPV	0.5 (0.5–0.6)	0.4 (0.4–0.5)	0.5 (0.4–0.5)	0.6 (0.5–0.6)
**Experienced health**				
- Very good	1.0		1.0	
- Good	1.1 (1.0–1.2)		1.5 (1.4–1.8)	
- Less than good	1.1 (1.0–1.3)		2.5 (2.1–2.9)	
**Suffering from a disease**				
- No	1.0	1.0	1.0	1.0
- Yes	1.2 (1.1–1.3)	1.1 (1.0–1.2)	2.0 (1.8–2.2)	1.3 (1.2–1.5)
Experienced quality of life (EQ5D tariff)**				
Per 10% decrease in score	1.04 (1.02–1.06)		1.19 (1.17–1.22)	1.08 (1.05–1.12)
**Change in experienced health**				
- No Change	1.0	1.0	1.0	1.0
- Better	1.1(1.0–1.2)	1.2 (1.0–1.3)	1.2 (1.0–1.4)	1.1 (0.9–1.4)
- Much better	0.8 (0.7–1.0)	0.8 (0.6–0.9)	1.3 (1.0–1.6)	1.2 (0.9–1.6)
- Worse	1.2 (1.1–1.3)	1.1 (1.0–1.2)	1.9 (1.7–2.1)	1.2 (1.1–1.4)
- Much worse	1.4 (1.2–1.7)	1.3 (1.1–1.6)	3.5 (2.9–4.3)	1.7 (1.4–2.2)
**Life-events**				
Did not experience life-event	1.0	1.0	1.0	1.0
Change in own health	1.3 (1.2–1.4)	1.2 (1.1–1.3)	1.8 (1.7–2.0)	1.4 (1.3–1.5)
Change in health of a loved-one	1.3 (1.2–1.4)	1.2 (1.1–1.3)	1.2 (1.1–1.3)	1.1 (1.0–1.3)
Death of a loved-one	1.3 (1.2–1.4)	1.4 (1.2–1.5)	1.2 (1.1–1.4)	1.2 (1.1–1.3)
Positive life-event	1.1 (1.0–1.3)		0.8 (0.7–0.9)	
Any life-event	1.3 (1.3–1.4)		1.5 (1.4–1.7)	

*For the analyses on communication using GEE, variables that were presented separately for the descriptive analyses in Table 2 were merged. ‘Talked with family’ consists of talking ‘With partner’, ‘With Children’ and ‘With other family members’. ‘Talked with physician’ consists of talked ‘With GP’ and ‘With medical specialist’.

**The EQ5D is a validated set of questions on five domains (mobility, self-care, usual activities, pain and discomfort, anxiety and depression), which can be used to calculate a single index value representing someone’s perceived quality of life. A score of 100% represents full health and a score of 0 represents death.

Having a partner, children, being female and having a belief that is of importance in one’s life increased the odds to talk with family. Higher age, having a higher education and a lower experienced quality of life (measured using the Eq5D), all increased the odds to discuss ADs with a physician. As was also seen in the descriptive figures, membership of the NPV makes it less likely to talk about ADs, with family as well as physicians.

## Discussion

A vast majority (ranging 96.9–98.1%) of people with an AD who experienced a life-event indicated their preferences remained unchanged or became stronger. This corresponds with earlier findings in research about this subject, showing that stability of preferences is higher among the owners of ADs [3,4,6,7,17]. Nonetheless this is a noteworthy result because we know that part of the experienced life-events, concerning serious health issues of a loved one, are important for the decision to draw up an AD [8]. Also changes in a person’s own health do not seem to influence the stability of preferences.

Many previous studies found no association between a change in health and instability of preferences [7,18–24]. From those that did find an association, some found that a change in health alters preferences towards wanting more treatment[3,6], while others found the opposite [2,25]. From this Auriemma et al. concluded in their review that the data on this subject was inconclusive [1]. Compared with the studies cited by Auriemma, ours had a longer follow-up, a bigger sample and the population consisted solely of people actually owning an AD. Our findings suggest no association between instability of preferences and the experience of life-events, including a change in health.

Of the NVVE-members who had experienced a life-event, the proportion making alterations in their AD was larger than the proportion with instable preferences. However, these percentages still represent small minorities (ranging 6.3–10.1%). Combined with our finding that preferences overall remained stable or became stronger, the alterations made in ADs could well be a sharpening of previous statements.

Another explanation for the difference between the number of instable preferences and alterations in ADs could be the fact that the NVVE published new versions of ADs during the course of our study. This may have been an incentive for respondents to draft a new one and is unrelated to instability of preferences. It could also explain the statistically significant differences between the 4 waves of questionnaires when it came to the percentages of respondents that made alterations in ADs (ranging 6.3–10.1%).

A third explanation of the difference between stability of preferences and alterations in ADs may be found in the way stability of preferences was assessed for this study. Respondents were asked if their views or preferences had changed in general, without specifying. We know from a study by Evans et al. that specific treatment preferences from elderly people do not always concur with their general end-of-life goals [26].

The majority of studies reviewed by Auriemma et al. assessed the stability of preferences by repeatedly asking about specific types of medical care over time [1]. This seems to be a more objective method as compared to the method used for this study. This has to be taken into account when interpreting the results of this study: the stability of preferences could be somewhat overestimated. However, in the context of ACP and palliative care in practice the self-perceived preference stability is highly relevant.

From the life-events only the experience of the death of a loved one remained significantly associated with alterations in ADs, next to a (subjectively reported) bad health and higher age among others.

People with an AD tend to talk about these documents, as shown by the fact that only small minorities indicated they did not talk at all about this subject during the six-year course of our study (8.7% for NVVE-members and 16.4% for NPV-members). However, taken into account that communication is essential to the success of ADs [9], there is still progress to be made, especially when it comes to the communication with physicians, which stays behind as compared to communication with loved ones. From qualitative studies we know that patients as well as caregivers experience barriers to discuss end-of-life preferences [8,27,28]. Talking about preferences may especially be relevant when there is a change in someone’s health. Our findings do show a change in health status is an incentive, because multiple variables linked to (a change in) health increased the odds to talk about ADs.

Strengths of our study are the long study period and the large number of cohort members. Our study also had some limitations. First, there may have been a recall bias when it comes to communication about ADs and alterations made in ADs. Second, our study in itself, by sending the questionnaires every one-and-a-half year, may have been an incentive for the respondents to think, talk about or make alterations in their ADs.

Third, although we were able to follow our respondents over time in order to assess stability of preferences and the experiences of life-events, this study doesn’t provide data about the end of life of the participants. We know from a study by Bischoff et al. that 37% of patients change their code status near the end of life [29]. On the other hand Bolt et al. found that a change or deterioration in health near the end of life generally did not seem to influence preferences concerning euthanasia of people with an advance euthanasia directive [30].

Finally, one can question to which extend the results of our study can be generalized to the general population, due to the fact that ADs in the Netherlands are not promoted by any policy and therefore are not widespread (only 7% of the Dutch population owns an AD)[11,12]. Results of previous studies we performed, where we compared the members of the cohort with the Dutch general public, showed both groups, NVVE- and NPV-members, can be placed at opposite end of the spectrum when it comes to views on then end of life in the Netherlands [10,14]. While not representative for the general public both groups at opposite ends of the spectrum show similar results.

## Conclusions

The results of our study make an important contribution to disputing the criticism concerning the usability of ADs due to a lack of stability of preferences of their owners. Whereas a change in health status and the experience of other life-events were not related to instability of general self-reported preferences about the end of life, they did increase the odds of communication about ADs. Yet, communication remains an issue of concern. In order to translate general preferences about end of life put down in ADs to specific decisions in practice, communication is vital. Our results show that possession of an AD does not necessarily lead to frequent discussions between patients and caregivers. A more structured approach, like ACP, might be useful to foster communication and improve the relevance of ADs in practice.

## Supporting information

S1 File Dataset used for the paper. SPSS-file, the data is structured long.
(SAV)

S2 File Questionnaire used to collect the data for the paper, original version in Dutch. (DOC)

S3 File Questionnaire used to collect the data for the paper, translated version in English. (DOC)
