# Supplementary material for: Stability of end-of-life preferences in relation to health status and life-events: A cohort study with a 6-year follow-up among holders of an advance directive
Source: PLoS One. 2018 Dec 18;13(12):e0209315. doi: 10.1371/journal.pone.0209315 (PMC6298688; doi:10.1371/journal.pone.0209315)
Supplement: S2 File — (DOC) [file pone.0209315.s002.doc]

| Lidnummer: |  |
| --- | --- |
| Verificatiecode: |  |

# VRAGENLIJST

##### WILSVERKLARINGEN

NVVE

| 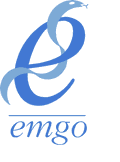 | 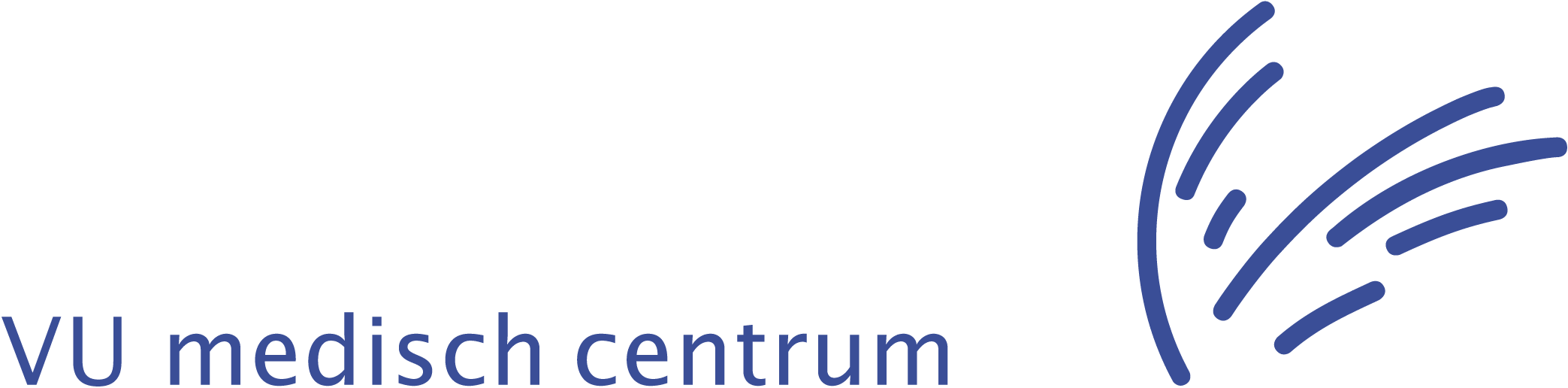 |
| --- | --- |

| **Enkele vragen over uw persoonlijke gegevens** | | |
| --- | --- | --- |
|  |  | |
| Wat is uw leeftijd? | ……… jaar | |
|  |  |  |
| U bent | ❑ Man❑ Vrouw | |
|  |  |  |
| Heeft u op dit moment een partner? | ❑ Ja, gehuwd ❑ Ja, samenwonend  ❑ Ja, anders  ❑ Nee, gescheiden  ❑ Nee, verweduwd  ❑ Nee, anders | |
|  |  | |
| Heeft u kinderen en hoe is het contact met die kinderen? | ❑ Ja, goed contact ❑ Ja, goed contact maar niet met allemaal  ❑ Ja, maar slecht of geen contact ❑ Nee, geen kinderen ❑ Anders, namelijk: ........................................………. | |
|  |  |  |
| Waar woont u? | ❑ Thuis ❑ Verzorgingshuis  ❑ Aanleunwoning  ❑ Verpleeghuis  ❑ Revalidatiecentrum ❑ Anders, namelijk: ........................................………. | |

| **Enkele vragen over uw gezondheid** |
| --- |

| Hoe is over het algemeen uw gezondheidstoestand? | | ❑ Zeer goed  ❑ Goed  ❑ Minder dan goed | | |
| --- | --- | --- | --- | --- |
|  |  | | |  |
| Hoe is uw gezondheidstoestand ten opzichte van anderhalf jaar geleden (dus sinds oktober 20..)? | | ❑ Veel beter  ❑ Iets beter ❑ Geen verschil ❑ Iets slechter  ❑ Veel slechter |  | |
|  |  | | |  |

| Heeft u een of meer van de volgende aandoeningen? (meerdere antwoorden mogelijk) | ❑ Nee  ❑ Ja, namelijk: ❑ Reuma/artrose❑ Astma/CARA/COPD ❑ Suikerziekte ❑ Hartaandoening ❑ (Gevolgen van) beroerte  ❑ Dementie  ❑ Multiple Sclerose (MS)  ❑ Amyotrofische Lateraal Sclerose (ALS)  ❑ Depressie  ❑ Anders, namelijk:………………………………  ................................................…....................... |
| --- | --- |

| Heeft u op dit moment een vorm van kanker (het gaat *niet* om kanker waarvan u genezen bent)? | ❑ Ja  ❑ Nee |
| --- | --- |

| Kunt u bij iedere groep in de lijst hieronder een kruisje zetten in het hokje voor de zin die het best past bij uw eigen gezondheidstoestand **vandaag**? |
| --- |

| 1. Mobiliteit   ❑ Ik heb geen problemen met lopen  ❑ Ik heb enige problemen met lopen  ❑ Ik zit in een rolstoel  ❑ Ik ben bedlegerig |
| --- |

| 1. Zelfzorg   ❑ Ik heb geen problemen om mijzelf te wassen of aan te kleden  ❑ Ik heb enige problemen om mijzelf te wassen of aan te kleden  ❑ Ik ben niet in staat mijzelf te wassen of aan te kleden |
| --- |

| 1. Dagelijkse activiteiten (bijv. werk, studie, huishouden, gezins- en vrijetijdsactiviteiten)   ❑ Ik heb geen problemen met mijn dagelijkse activiteiten  ❑ Ik heb enige problemen met mijn dagelijkse activiteiten  ❑ Ik ben niet in staat mijn dagelijkse activiteiten uit te voeren |
| --- |

| 1. Pijn en andere klachten   ❑ Ik heb geen pijn of andere klachten  ❑ Ik heb matige pijn of andere klachten  ❑ Ik heb zeer ernstige pijn of andere klachten |
| --- |

| 1. Stemming   ❑ Ik ben niet angstig of somber  ❑ Ik ben matig angstig of somber  ❑ Ik ben erg angstig of somber |
| --- |

| 1. Eenzaamheid   ❑ Ik ben niet eenzaam  ❑ Ik ben een beetje eenzaam  ❑ Ik ben erg eenzaam |
| --- |

| **Vervolgens enkele vragen over veranderingen in ervaringen en wensen of opvattingen over het levenseinde en in uw persoonlijke situatie.** | |
| --- | --- |
|  |  |
| Kunt u hiernaast aangeven of u de volgende gebeurtenissen heeft meegemaakt in de afgelopen anderhalf jaar (dus sinds oktober 20..)? (meerdere antwoorden mogelijk) | ❑ Géén gebeurtenissen, zoals hieronder genoemd(ga door naar vraag ..) De volgende gebeurtenissen: **Gezondheid** (zowel geestelijk als lichamelijk, bijvoorbeeld de diagnose, behandeling of genezing van een ziekte)❑ Verandering van mijn eigen gezondheid❑ Verandering van de gezondheid van mijn partner❑ Verandering van de gezondheid van een andere dierbare,namelijk: ……………………………………………………….**Familie/woonsituatie**❑ Ik heb een (klein)kind gekregen ❑ Ik heb een nieuwe partner/vriend(in)  ❑ Ik ben verhuisd naar een verzorgingshuis  ❑ Ik ben verhuisd naar een verpleeghuis  ❑ Ik ben verhuisd naar een andere plek  Overlijdens ❑ Verlies van mijn partner❑ Verlies van een andere dierbare, namelijk:…………………………………………..…………………………**Anders**❑ Andere gebeurtenissen namelijk:…………………………… ………………………………………………..……………………  ……………………………………..……………………………… |

| Hebben deze gebeurtenissen geleid tot veranderingen in uw opvattingen of wensen over het levenseinde ten opzichte van anderhalf jaar geleden (dus sinds oktober 20..)? | ❑ Nee, mijn opvattingen en wensen zijn onveranderd  ❑ Ja, mijn al bestaande opvattingen en wensen zijn versterkt  ❑ Ja, ik ben gaan twijfelen over bepaalde opvattingen en wensen  ❑ Ja, mijn opvattingen en wensen zijn inhoudelijk veranderd | | |
| --- | --- | --- | --- |
|  | |  |  |

| **Vragen over wilsverklaringen** |
| --- |

| Heeft u sinds anderhalf jaar geleden (dus sinds oktober 20..) nieuwe wilsverklaringen ingevuld of wijzigingen aangebracht in uw wilsverklaringen? (meerdere antwoorden mogelijk) | ❑ Nee (ga door naar vraag ..)  ❑ Ja, ik heb de volgende wilsverklaring(en) **voor het eerst ingevuld**:  ❑ Euthanasieverzoek  ❑ Behandelverbod  ❑ Volmacht  ❑ Bijzondere clausule 'Voltooid leven'  ❑ Niet-reanimerenpenning  ❑ Bijsluiter Europees medisch paspoort |
| --- | --- |

|  | ❑ Ja, ik heb **wijzigingen** aangebracht in de volgende wilsverklaring(en):  ❑ Euthanasieverzoek  ❑ Behandelverbod  ❑ Volmacht  ❑ Bijzondere clausule 'Voltooid leven'  ❑ Niet-reanimerenpenning |
| --- | --- |
|  | Ik heb de volgende wijzigingen aangebracht in mijn wilsverklaringen  …………………………………………………………  ………………………………………………………… |

| Op welke datum heeft u deze wilsverklaring(en) ingevuld/gewijzigd? (Zo nodig meerdere data invullen.) | ……………………………(maand, jaar) |
| --- | --- |

| Heeft u in het afgelopen anderhalf jaar (dus sinds oktober 20..) met anderen over uw wilsverklaringen gesproken? (meerdere antwoorden mogelijk) | ❑ Nee  ❑ Ja, met mijn partner  ❑ Ja, met mijn kinderen  ❑ Ja, met mijn huisarts  ❑ Ja, met mijn specialist  ❑ Ja, met anderen namelijk:………………………..…… |
| --- | --- |
