# Supplementary material for: Stability of end-of-life preferences in relation to health status and life-events: A cohort study with a 6-year follow-up among holders of an advance directive
Source: PLoS One. 2018 Dec 18;13(12):e0209315. doi: 10.1371/journal.pone.0209315 (PMC6298688; doi:10.1371/journal.pone.0209315)
Supplement: S3 File — (DOC) [file pone.0209315.s003.doc]

| ID-number: |  |
| --- | --- |
|  |  |

# QUESTIONNAIRE

##### ADVANCE DIRECTIVES

NVVE

| 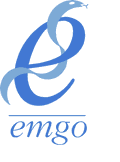 | 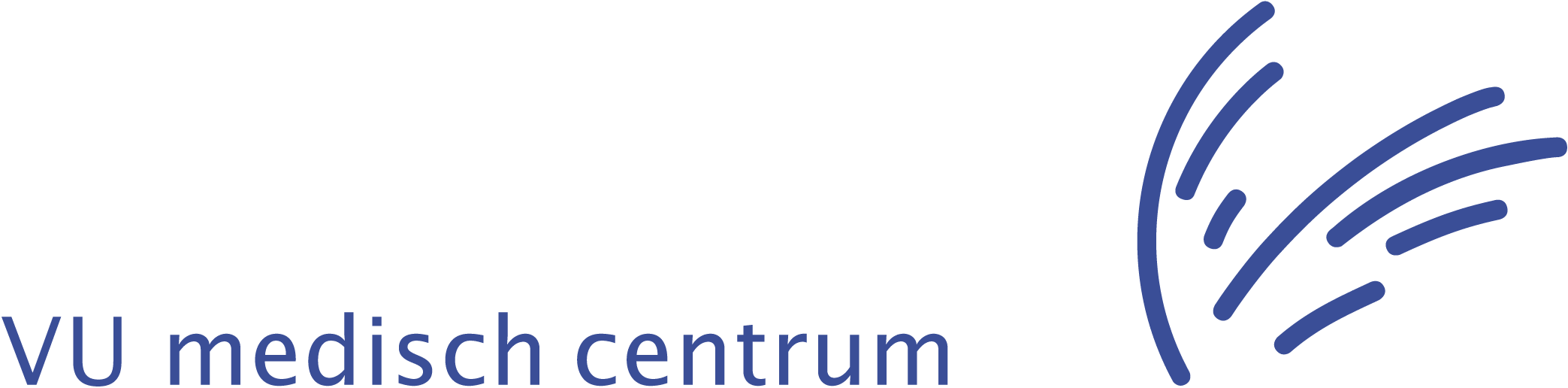 |
| --- | --- |

| **Some questions about your personal background** | | |
| --- | --- | --- |
|  |  | |
| What is your age? | ……… years | |
|  |  |  |
| You are | ❑ Male❑ Female | |
|  |  |  |
| Do you have a partner at the moment? | ❑ Yes, married ❑ Yes, living together  ❑ Yes, otherwise  ❑ No, divorced  ❑ No, widowed  ❑ No, otherwise | |
|  |  | |
| Do you have children en how is your contact with them? | ❑ Yes, good contact ❑ Yes, good contact but not with all  ❑ Yes, but bad or no contact ❑ No, no children ❑ Otherwise, namely: ........................................………. | |
|  |  |  |
| Where do you live? | ❑ Own home ❑ Home for elderly  ❑ Sheltered living  ❑ Nursing-home  ❑ Rehabilitation centre ❑ Otherwise, namely: ........................................………. | |

| **Some questions about your health** |
| --- |

| How is generally your health status? | | ❑ Very well  ❑ Well  ❑ Less than well | | |
| --- | --- | --- | --- | --- |
|  |  | | |  |
| How is you health status compared to 1,5 years prior (so since October 20..)? | | ❑ Much better  ❑ Somewhat better ❑ No difference ❑ Somewhat worse  ❑ Much worse |  | |
|  |  | | |  |

| Do you suffer from one or more of these diseases? (more than one answer possible) | ❑ No  ❑ Yes, namely: ❑ Rheumatism/artrosis❑ Asthma/COPD ❑ Diabetes ❑ Cardiac condition ❑ (Consequences of a) stroke  ❑ Dementia  ❑ Multiple Sclerosis (MS)  ❑ Amyotrophic Lateral Sclerosis (ALS)  ❑ Depression  ❑ Otherwise, namely:………………………………  ................................................…....................... |
| --- | --- |

| Do you suffer from a form of cancer at the moment (not meaning cancer that you are cured from)? | ❑ Yes  ❑ No |
| --- | --- |

| Could you check the box at the sentence that best describes your health status **today** at each subject in the list below? |
| --- |

| 1. Mobility   ❑ I have no problems walking  ❑ I have some problems walking  ❑ I am in a wheelchair  ❑ I am bedridden |
| --- |

| 1. Self-care   ❑ I have no problems washing or dressing myself  ❑ I have some problems washing or dressing myself  ❑ I am not able to wash or dress myself |
| --- |

| 1. Daily activities (e.g. work, study, household, family- and leisure-time activities)   ❑ I have no problems performing my daily activities  ❑ I have some problems performing my daily activities  ❑ I am not able to perform my daily activities |
| --- |

| 1. Pain and other symptoms   ❑ I have no pain or other symptoms  ❑ I have moderate pain or other symptoms  ❑ I have severe pain or other symptoms |
| --- |

| 1. Mental state   ❑ I am not anxious or depressed  ❑ I am moderately anxious or depressed  ❑ I am very anxious or depressed |
| --- |

| 1. Loneliness   ❑ I am not lonely  ❑ I am a bit lonely  ❑ I am very lonely |
| --- |

| **Subsequently some questions about changes in experiences and preferences or views about the end of life and in you personal situation.** | |
| --- | --- |
|  |  |
| Could you indicate here if you experienced one of the following events the past one and a half year? (so since October 20..)? (more than one answer possible) | ❑ No events as mentioned below(precede to question ..) The following events: **Health** (both mental or physical, for instance the diagnosis, treatment or recovery of a disease)❑ Change in my own health❑ Change in the health of my partner❑ Change in the health of another close-one,namely: ……………………………………………………….**Family/situation of living**❑ I got a (grand)child ❑ I got a new partner/boy- or girlfriend  ❑ I moved to a home for the elderly  ❑ I moved to a nursing-home  ❑ I moved to a different place  Deaths ❑ Loss of my partner❑ Loss of another close-one, namely:…………………………………………..…………………………**Otherwise**❑ Other events namely:…………………………… ………………………………………………..……………………  ……………………………………..……………………………… |

| Did these events lead to changes in your views or preferences about the end of life as compared to 1,5 years prior (so since October 20..)? | ❑ No, my views and preferences are unchanged  ❑ Yes, my existing views and preferences became stronger  ❑ Yes, I am in doubt about certain views and preferences  ❑ Yes, my views and preferences have changed substantively | | |
| --- | --- | --- | --- |
|  | |  |  |

| **Questions about advance directives** |
| --- |

| Have you completed new advance directives since one and a half years prior (so since October 20..)? (more than one answer possible) | ❑ No (precede to question ..)  ❑Yes, I completed the following advance directives **for the first time**:  ❑Request for euthanasia  ❑Refusal of treatment document  ❑Healthcare proxy  ❑Special clause ‘Completed life'  ❑Do not resuscitate order |
| --- | --- |

|  | ❑Yes, I made **alteration**s in the following advance directive(s):  ❑Request for euthanasia  ❑Refusal of treatment document  ❑Healthcare proxy  ❑Special clause ‘Completed life'  ❑Do not resuscitate order |
| --- | --- |
|  | I made the following alterations in my advance directives  …………………………………………………………  ………………………………………………………… |

| At which date did you complete/change the advance directive(s)? (If necessary fill in more than one date.) | ……………………………(month, year) |
| --- | --- |

| Did you talk with others about your advance directive the past one and a half years (so since October 20..)? (more than one answer possible) | ❑ No  ❑ Yes, with my partner  ❑ Yes, with my children  ❑ Yes, with my general practitioner  ❑ Yes, with my medical specialist  ❑ Yes, with others namely:………………………..…… |
| --- | --- |
